# Supplementary material for: Siponimod Attenuates Neuronal Cell Death Triggered by Neuroinflammation via NFκB and Mitochondrial Pathways
Source: Int J Mol Sci. 2024 Feb 20;25(5):2454. doi: 10.3390/ijms25052454 (PMC10931690; doi:10.3390/ijms25052454)
Supplement: Supplementary file 1 [file ijms-25-02454-s001.zip › ijms-2843509-supplementary.pdf]

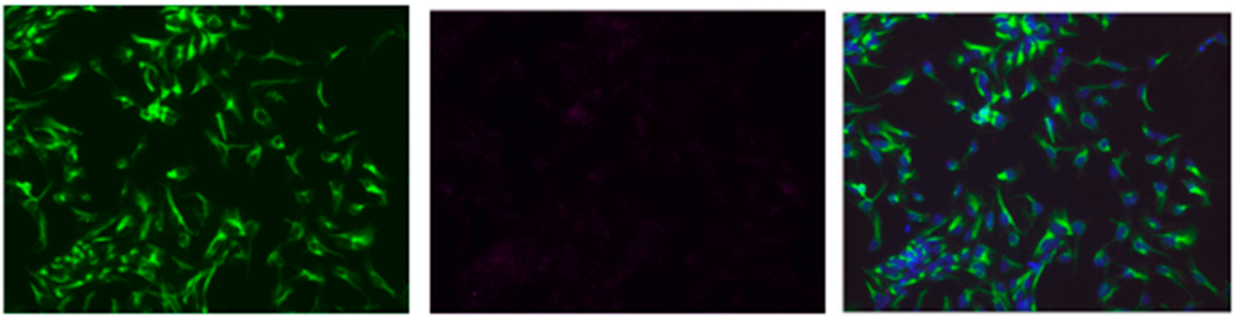

Supplementary Figure S1. **Generation of human neuronal hiPSC-NSCs/NPCs cell line**. Nestin was used as marker for NPCs (green). Lack of Oct-3/4 (red) staining indicated that no remnant iPSCs were present in the culture (almost 100% NSC/NPC). DAPI (blue) stained for nucleus.

A

B

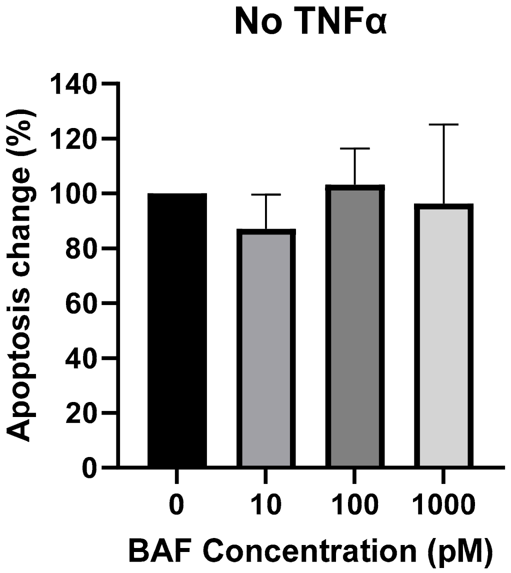
No TNFα
Apoptosis change (%)
140
120
100
80
60
40
20
0
0
10
100
1000
BAF Concentration (pM)

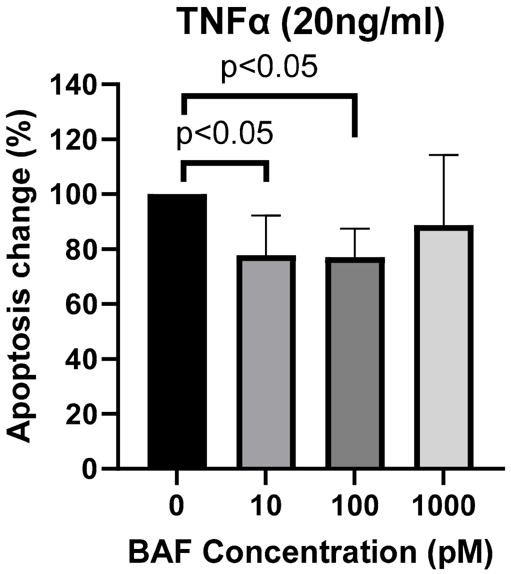
TNFα (20ng/ml)
p<0.05
p<0.05
Apoptosis change (%)
140
120
100
80
60
40
20
0
0
10
100
1000
BAF Concentration (pM)

C

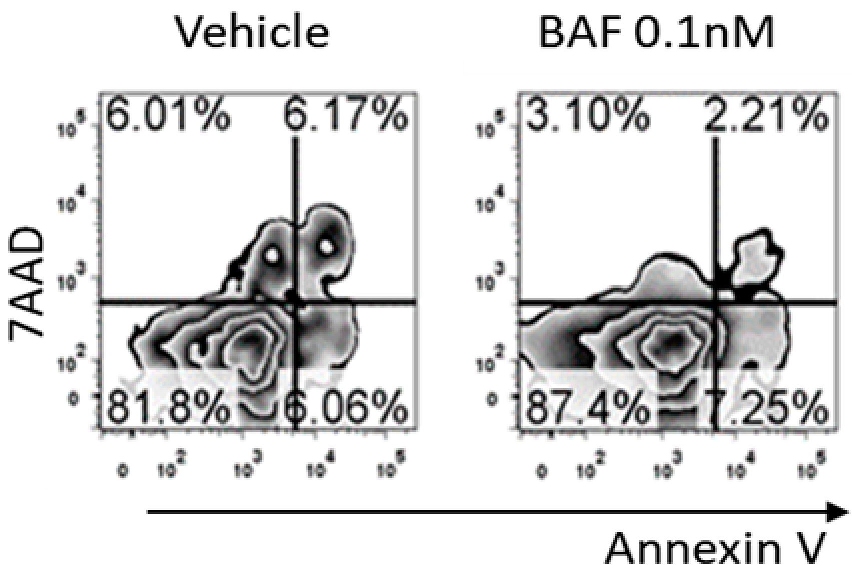

Supplementary Figure S2. Siponimod/BAF312 reduced apoptosis of human neuronal hiPSCs-NSCs/NPCs cells (Annexin V+7AAD-%) based on flowcytometry. Human neuronal hiPSCs-NSCs/NPCs cells treated with Siponimod/BAF312 (abbreviated as BAF) at concentrations of 0, 10, 100, 1000 pM under with **(A)** or without **(B)** TNFα (20ng/ml) treatment for 48h. These experiments were done in duplicate. The average of the duplicate was calculated as fraction of average of the vehicle control (DMSO) in that experiment. The average from four experiments of these fractions were presented as column with SD. Relative cell death or survival was calculated compared to relevant vehicle controls (DMSO). A one-way ANOVA test was used to compare changes in apoptosis between concentrations of Siponimod/BAF312 (BAF). P values <0.05 were considered significant. **(C).** Representative flow plots from three independent experiments.

A

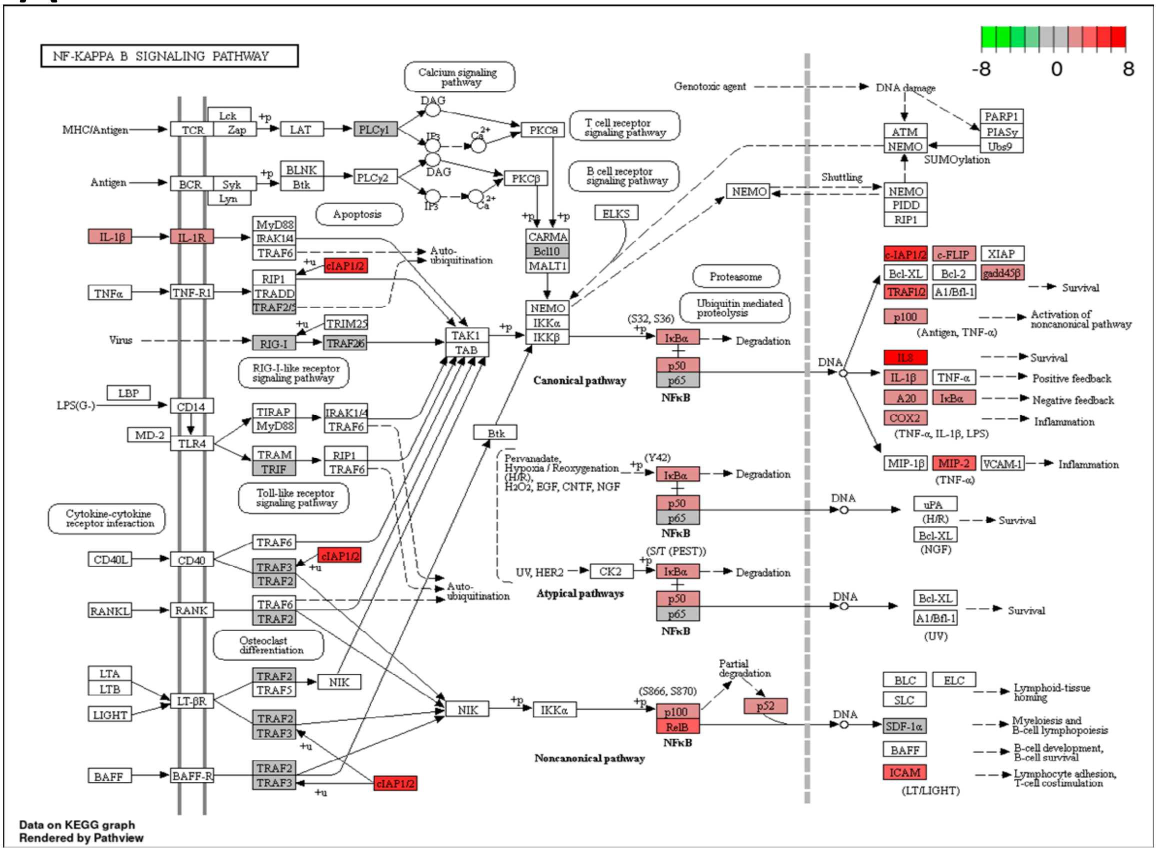
NF-KAPPA B SIGNALING PATHWAY
-8
0
8
MHC/Antigen
TCR
Lck
Zap
LAT
PLCγ1
DAG
PKCθ
Calcium signaling pathway
T cell receptor signaling pathway
B cell receptor signaling pathway
Antigen
BCR
Syk
Lyn
BLNK
Btk
PLCγ2
PKCβ
Apoptosis
IL-1β
IL-1R
MyD88
IRAK1/4
TRAF6
cIAP1/2
Auto-ubiquitination
TNFα
TNF-R1
RIP1
TRADD
TRAF2/5
Virus
RIG-I
TRIM25
TRAF3
TRAF6
RIG-I-like receptor signaling pathway
LBP
LPS(G-)
CD14
MD-2
TLR4
TIRAP
MyD88
IRAK1/4
TRAF6
TRAM
TRIF
RIP1
TRAF6
Toll-like receptor signaling pathway
Cytokine-cytokine receptor interaction
CD40L
CD40
TRAF6
TRAF3
TRAF2
cIAP1/2
RANKL
RANK
TRAF6
TRAF2
Osteoclast differentiation
LTA
LTB
LIGHT
LT-βR
TRAF2
TRAF5
NIK
TRAF2
TRAF3
BAFF
BAFF-R
TRAF2
TRAF3
cIAP1/2
CARMA
Bcl10
MALT1
ELKS
NEMO
IKKα
IKKβ
TAK1
TAB
Btk
Proteasome
Ubiquitin mediated proteolysis
(S32, S36)
IκBα
Degradation
p50
p65
NFκB
Canonical pathway
Pervanadate, Hypoxia / Reoxygenation (H/R), H2O2, EGF, CNTF, NGF
(Y42)
IκBα
Degradation
p50
p65
NFκB
UV, HER2
CK2
(S/T (PEST))
IκBα
Degradation
p50
p65
NFκB
Atypical pathways
NIK
IKKα
(S866, S870)
p100
RelB
NFκB
Partial degradation
p52
Noncanonical pathway
Genotoxic agent
DNA damage
ATM
NEMO
PARP1
PIASy
Ubc9
SUMOylation
Shuttling
NEMO
NEMO
PIDD
RIP1
DNA
c-IAP1/2
c-FLIP
XIAP
Bcl-XL
Bcl-2
gadd45β
Survival
TRAF1/2
A1/Bfl-1
p100
(Antigen, TNF-α)
Activation of noncanonical pathway
IL8
Survival
IL-1β
TNF-α
Positive feedback
A20
IκBα
Negative feedback
COX2
Inflammation
(TNF-α, IL-1β, LPS)
MIP-1β
MIP-2
VCAM-1
Inflammation
(TNF-α)
uPA
(H/R)
Bcl-XL
(NGF)
Survival
Bcl-XL
A1/Bfl-1
(UV)
Survival
BLC
ELC
SLC
SDF-1α
BAFF
ICAM
(LT/LIGHT)
Lymphoid-tissue homing
Myeloiesis and B-cell lymphopoiesis
B-cell development, B-cell survival
Lymphocyte adhesion, T-cell costimulation
Data on KEGG graph
Rendered by Pathview

B

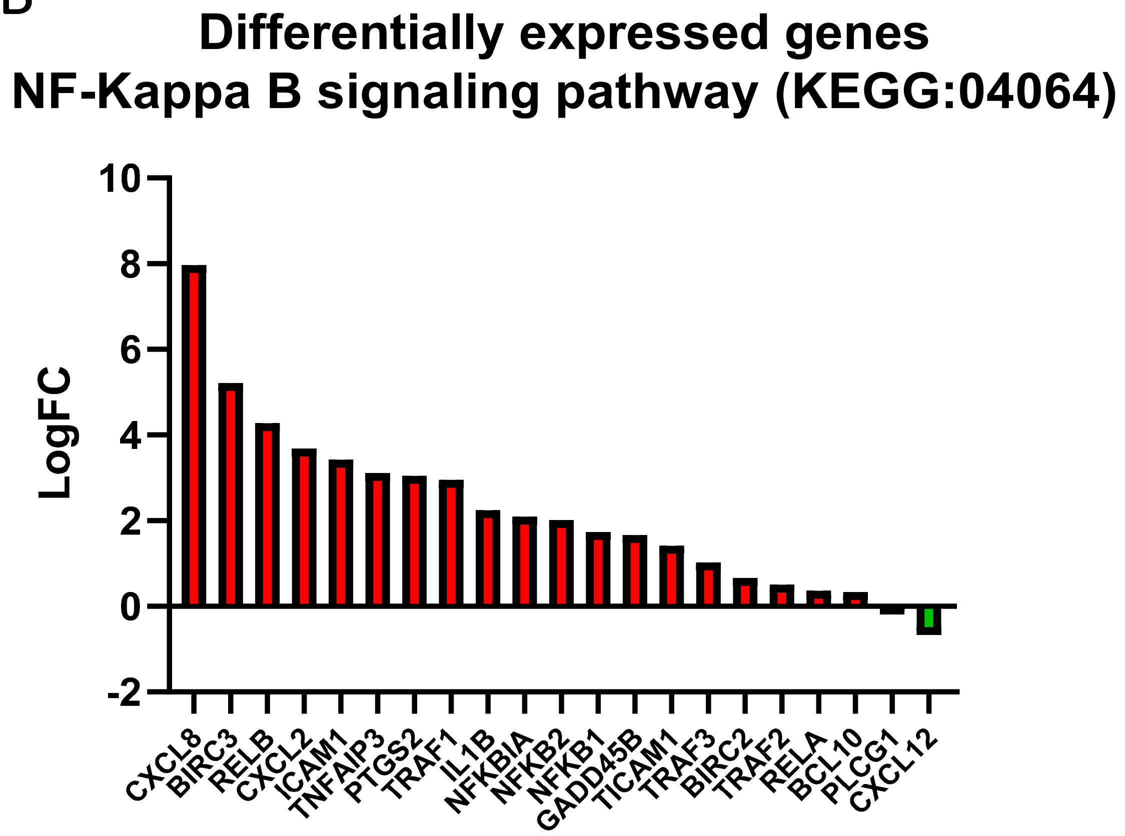
Differentially expressed genes
NF-Kappa B signaling pathway (KEGG:04064)
LogFC
10
8
6
4
2
0
-2
CXCL8
BIRC3
RELB
CXCL2
ICAM1
TNFAIP3
PTGS2
TRAF1
IL1B
NFKBIA
NFKB2
NFKB1
GADD45B
TICAM1
TRAF3
BIRC2
TRAF2
RELA
BCL10
PLCG1
CXCL12

Supplementary Figure S3. TNFα treatment affects genes involved in NFKB signaling pathways in human neuronal hiPSCs-NSCs/NPCs cells. (**A**) The NFKB pathway (KEGG: 04064) diagram is overlaid with the expression changes of each gene. Downregulated genes are shown in green, while upregulated genes are in red. (**B**) The top 20 differentially expressed genes in NFKB signaling pathway (KEGG: 04064) are ranked based on their absolute value of log fold change. Upregulated genes are shown in red, and downregulated genes are shown in green. *n* = 5-6 sets.
